# Round the Hospitals

**Published:** 1917-03-10

**Authors:** 


### ROUND THE HOSPITALS.

Miss Ruth E. Darbyshire, R.R.C., relinquished her duties at St. Mary's and left the hospital on the 2nd instant. On the 28th ultimo she was photographed with the entire staff of St. Mary's nurses and also, by special request, with all the St. Mary's sisters. Miss Darbyshire has been the recipient of several presentations. Princess Arthur of Connaught gave her a travelling clock; the nursing staff of St. Mary's a handsome natural musquash coat; a wounded soldier, Private Purvis, worked and presented to her a dainty bead bag which was much admired; and a group of nurses temporarily engaged for war work a pair of silver shoe-buckles. On an earlier day, in the previous week, the nursing staff of the 2nd London General Hospital presented Miss Darbyshire with a complete set of ivory toilet accessories, a replica of the Royal Red Cross, and a luncheon-basket, in addition to a leather-bound volume in which the signatures of the whole nursing staff were entered, with a covering letter. The badge of the Territorial Force Nursing Service constituted the frontispiece, and the volume contained a reproduction of the Royal Red Cross, the date of its presentation by the King, and the date of Miss Darbyshire's resignation of the Principal Matronship. Miss Riddell, the Matron of the 2nd London General Hospital, made the presentations in a well-conceived speech, in the course of which she emphasised the happy relations which had existed between the Principal Matron, the Matron, and all the staff. Miss Lloyd, one of the V.A.D. workers, presented a bouquet of carnations and lilies of the valley from the staff, wishing her every happiness, and Miss Pearce, one of the sisters who was mobilised in 1914, referred to the early days of the war when all the workers in the hospital were strangers to each other. Miss Darbyshire, she declared, took broad-minded views and quickly united all ranks behind her, so that the work had been carried on in perfect harmony from the outset. Miss Darbyshire, in reply, spoke with grateful appreciation of Miss Riddell's administrative gifts, and declared her time at the 2nd London General Hospital to have been a very happy one, without one rift in the lute. These presentations were notable for the large attendances at each, and the harmonious heartiness and satisfaction which characterised the whole proceedings from first to last.

March has come, and with it the majority of matrons throughout the country have before them, or in a position approaching completion, the summary of their nursing staff for 1916. We shall be very glad, having regard to the importance of a correct history and record being maintained year by year, to receive from each matron the summary of her staff for 1916. For upwards of thirty years The Hospital has continuously published particulars of progress in the matrons' departments and the development of British nurse-training schools throughout the country, so that it has become especially the matron's organ which many of the most experienced and knowledgeable regard as their friend and helper in the press. The summary for 1916 should have special interest. It will in the future have an historical value, relating as it does to what, owing to the war, has probably been the most difficult year British matrons have had to face. We shall look forward to the receipt of the summary of the nursing staff not only of the voluntary, but of the military and territorial hospitals, that full justice may be done to the self-denying and splendid work accomplished for the Nation and Empire by Homeland workers throughout our hospital system. It is indeed matter for congratulation in this connection, that Miss Margaret Thomas, who has been on the staff of the Royal Infirmary, Liverpool, for thirty years and is at present acting sister in one of the military wards, has been awarded the Royal Red Cross decoration. We congratulate those charged with the onerous duty of selecting devoted workers for decorations upon the award of the Royal Red Cross to Acting Sister Margaret Thomas.

---

We have received the Report for 1916 of Miss Margaret Cummins, the Matron of the Liverpool Royal Infirmary. Although the chances of promotion in this hospital make the larger appeal to its *alumnæ*, it is noteworthy that the majority of the nurses who have completed their training in wartime have joined the Q.A.I.M.N.S.R. The hospital staff at the beginning of 1917 numbered 106. Thirty-five nurses entered for training, nine of whom left on the expiration of the trial period and twenty-three are completing their training. Of twenty-two nurses who have left, twelve were transferred to military nursing, two to district

**ROUND THE HOSPITALS** *(continued)*.

nursing, four were appointed sisters in the Northampton General Hospital, one joined the A.P.M.M.C., and three have been married. The private staff numbers twenty, who nursed 127 cases during the year. Notwithstanding the retirement of several nurses from military service, forty-four are still engaged in naval or military work, chiefly abroad. Amongst the notable honours gained by the nursing staff are: Nurse Rosetta Miller, awarded the Royal Red Cross decoration, who has been twice mentioned in dispatches, whilst Nurses Elsie Garner and Elsie Johnson, both acting-sisters on the Western Front, have also been mentioned in dispatches. Miss Atkinson, after being senior home sister and matron's assistant at Liverpool, was appointed matron of the Northampton General Hospital in March 1916. She was a distinct loss to the nursing department of the Royal Infirmary, Liverpool, and one of the sisters went with her as housekeeping sister at Northampton. It is evidence of the strength and efficiency of the nurse-training at the Royal Infirmary, Liverpool, that promotions to the higher posts are made from the nurses trained there. In 1916, amongst other appointments, may be specially mentioned that of Sister Crichton to the post of senior home sister, of Sister Mary Williams as night sister, and Sister Harkness as second home sister. Another Liverpool nurse, Miss MacInnes, obtained an appointment in a Belgian hospital after three years' service in the Liverpool Home, where she was succeeded as senior home sister by Miss Mary Jones. Seven nurses have been promoted to sisters' posts, of whom one has since accepted a post in India, and three are attached to military hospitals. The health of the nurses has been good on the whole, for although seventy-eight were off duty during the year there was not one serious case amongst them. The matron is to be congratulated on the excellent spirit which characterises the whole of the nursing staff at Liverpool, where various changes in the dietary, including margarine in the place of butter, and the cutting down at intervals of small luxuries, have been accepted in the right spirit, with an entire absence of grumbling.

Four columns of closely printed matter in the *Times* of the 5th instant are occupied by the names of ladies who have been brought to the notice of the Secretary of State for War for valuable services rendered in connection with the war. This is the first list of the kind which the present generation has seen, and it marks the extreme value attached to the services of all ranks of women workers who have ministered to the sick and wounded during the present war. The list includes every branch of service rendered by women in this connection, from Miss Becher, Matron-in-Chief Q.A.I.M.N.S., and Miss Sidney Browne, Matron-in-Chief T.F.N.S., to many matrons, sisters, staff and probationer nurses, Almeric Paget Military Massage Corps members, Commandants, General Service superintendents, nursing sisters S.J.A.B., V.A.D. nurses, military probationers, probationers asylum staff, special probationers, nursing V.A.D.s, nursing, probationer, and worker V.A.D.s and probationers W.H.C. These matrons, sisters, nurses, probationers and V.A.D.s have been attached to civil, war, military, territorial, Red Cross, auxiliary, infectious and cottage hospitals, there are also nurses from the Darlington Urban Infirmary and other infirmaries and convalescent homes, in addition to members of various Nursing Services, both home and colonial. We suggest that the matrons and home sisters throughout the country should make a point of obtaining a copy of the *Times* for March 5, 1917, that they may cut out page 6, paste it on a cardboard and suspend this wonderful record of nurses' service in Home Hospitals, which will become of increasing interest year by year and cannot fail to go down to history as evidence of the fine spirit, courage, and patriotism of British women at the time of the Great War.

